# A XGBoost-Based Prediction Method for Meat Sheep Transport Stress Using Wearable Photoelectric Sensors and Infrared Thermometry

**DOI:** 10.3390/s24237826

**Published:** 2024-12-07

**Authors:** Ruiqin Ma, Runqing Chen, Buwen Liang, Xinxing Li

**Affiliations:** 1 National Research Facility for Pheontypic and Genotypic Analysis of Model Animals (BEIJING), China Agricultural University, Beijing 100083, China; 2 College of Information and Electrical Engineering, China Agricultural University, Beijing 100083, China

**Keywords:** meat sheep, transport stress, biosensing, wearable IoT, machine learning

## Abstract

Transportation pressure poses a serious threat to the health of live sheep and the quality of their meat. So, the edible Hu sheep was chosen as the research object for meat sheep. We constructed a systematic biosignal detecting, processing, and modeling method. The biosignal sensing was performed with wearable sensors (photoelectric sensor and infrared temperature measurement) for physiological dynamic sensing and continuous monitoring of the transport environment of meat sheep. Core waveform extraction and modern spectral estimation methods are used to determine and strip out the target signal waveform from it for the purpose of accurate sensing and the acquisition of key transport parameters. Subsequently, we built a qualitative stress assessment method based on external manifestations with reference to the Karolinska drowsiness scale to establish stage classification rules for monitoring data in the transportation environment of meat sheep. Finally, machine learning algorithms such as Gaussian Naive Bayes (GaussianNB), Passive-Aggressive Aggregative Classifier (PAC), Nearest Centroid (NC), K-Nearest Neighbor Classification (KNN), Random Forest (RF), Support Vector Classification (SVC), Gradient Boosting Decision Tree (GBDT), and eXtreme Gradient Boosting (XGB) were established to predict the classification models of transportation stress in meat sheep. Their classification results were compared. The results show that SVC and GBDT algorithms are more effective and the overall model classification accuracy reached 86.44% and 91.53%. XGB has the best results. The accuracy of the assessment of the transport stress state of meat sheep after the optimization of three parameters was 100%, 90.91%, and 93.33%, and the classification accuracy of the overall model reached 94.92%. The final results achieved improve transport reliability, reduce transport risk, and solve the problems of inefficient meat sheep transport supervision and quality control.

## 1. Introduction

Mutton is a nutritious food source, and the per capita consumption of mutton in China has risen to 1.5 kg over the past few years, exceeding the world average. The rapidly growing demand for mutton not only stimulates the development of the meat and sheep industry, but also raises the quality and safety standards for mutton. Live sheep transportation is an essential step in the meat production market. However, transport-induced stress poses a critical challenge to the health of live sheep and the quality of meat. The key issue in sheep logistics is monitoring and assessing transport-induced stress to ensure the health of sheep, improve survival rates, and reduce distribution costs.

The use of wearable sensing technology in poultry breeding and the transportation industry is becoming more widespread. Properly constructed and utilized, wearable sensors can monitor poultry body temperature and heart rate parameters in real time, helping to prevent poultry diseases and minimize financial losses.

Mentis et al. [1] present some of the AI and ML applications for solving biomedical issues related to psychological stress, proving that AI and ML have been able to predict stress and detect the brain’s normal states vs. abnormal states with around 90% accuracy. Wei Wang et al. [2] conducted a systematic review and meta-analysis to identify, classify, and evaluate the evidence for novel wearable and non-contact devices that measure heart rate, respiratory rate, and oxygen saturation in the clinical setting. Mitar Simić et al. [3] developed a methodology for the realization of wearable, rechargeable battery-powered, small-sized, electronic devices for recording electromyogram (EMG) signals, suggesting that multiday continuous functionality is possible. Additionally, they combined the customizable EMG recording system with an efficient cross-correlation-based algorithm to monitor contractions/relaxations of muscles. Weiwei Jiang et al. [4] developed a neural network boosting regression model (i.e., NNBoost) and demonstrated that NNBoost outperforms other machine learning algorithms. Buwen Liang et al. [5] developed an ensemble learning strategy, synthesizing the benefits of Linear, SVR, MLP, KNN, Gaussian Process, and Decision Tree Algorithms, to detect salmon freshness and shelf-life and coupled sensors with stacking ensemble learning algorithms, achieving a data-driven, cost-effective, and efficient approach. Ruiqin Ma et al. [6] established the graded prediction model of meat sheep transportation stress by GA-BPNN and compared the performance it with the BP neural network optimized by particle swarm algorithm, simulated annealing algorithm, and ant colony algorithm, and the average prediction accuracy of the model was able to reach 89.81%.

The stimuli are called stressors, and physiological and behavioral changes in response to exposure to stressors constitute the stress response [7]. The stress response is divided into three phases: the alarm phase, the adaptation (or resistance) phase, and the exhaustion phase. During the alarm phase, the body activates all defense mechanisms to counter the adverse effects of stress. The adaptation phase features a reduction in or disappearance of the initial reaction symptoms. If the organism is exposed to the stressor for an extended period, it loses this acquired adaptability and enters the exhaustion phase, where the animal may ultimately face death unless emergency assistance is provided from an external source [8].

The successful transportation of live sheep requires highly efficient biosensing technology. However, existing live sheep transportation management and technology do not have consistent quality and safety protocols, resulting in limited technological advancement in equipment for live sheep preservation during transportation. Furthermore, traditional transportation systems primarily focus on monitoring single environmental factors, such as temperature, and fail to meet the requirements for thorough real-time physiological monitoring of live sheep. Most existing studies focus on the static detection of animal stress responses, which exhibit gradual signal variations, leading to poor monitoring continuity, high power consumption of detection devices, and the inability to continuously monitor livestock during breeding and transportation.

Consequently, this paper conducts a study on the health management and control strategies for stress in live sheep during meat transportation. We applied user-friendly and cost-effective sensor technology as a biosensing tool to develop a machine learning-based detection and assessment model for sheep transportation. This model provides crucial technical and management support for establishing a food safety assurance system in China.

In this paper, we adopted a “stress detection–expert evaluation–grading prediction” framework and focused on the Lake sheep breed as our research object. The main contributions are as followed:

- We developed a systematic method for detecting, processing, and modeling biosignals to accurately achieve sense and acquire key transportation parameters.
- We established a qualitative stress assessment method based on external performance, along with a graded prediction model utilizing various machine learning algorithms, to create a structured management mechanism for sheep transportation stress.
- We compared the prediction accuracies of several algorithms and identified the three with the highest accuracy: Support Vector Machine (SVM), Gradient Boosting Decision Tree (GBDT), and eXtreme Gradient Boosting (XGB) models.
- This approach enhances the reliability of transportation, reduces the associated risk, addresses inefficiencies in meat sheep transportation supervision and quality control, and provides essential technical and management support for establishing China’s food safety assurance system.

## 2. Materials and Methods

### 2.1. Data

A total of 195 sheep were used for modeling and evaluation in this study. All experimental protocols received approval from the Animal Ethics and Use Committee of China Agricultural University.

#### 2.1.1. Data Acquisition

In this section, we will outline the construction of biosignal dynamic detection, waveform extraction, and pre-processing. The biosignal dynamic monitoring consists of two main components: pulse wave signal detection and body temperature signal detection. The pulse wave signal itself is further divided into five components, including heart rate variability. The biosignal perception modeling involves the acquisition of photoplethysmographic (PPG) and electrocardiogram (ECG) signals [9], as well as a model for far-infrared body temperature perception.

(1) PPG ECG signal acquisition

The detection principle of the photo volumetric tracing method measures pulse and oxygen saturation through different light transmission rates in sheep tissue, caused by vascular pulsation.

The photoelectric sensor is placed on the exposed skin below the neck of a live sheep using a strap or clip. When the light beam illustrates the peripheral blood vessels in the sheep’s epidermis, the photoelectric converter detects the reflected data from the skin cells and transforms them into an electrical signal. This electrical signal is amplified and transmitted to the controller. The pulse signal varies periodically with the heartbeat, and the volume of the arterial blood vessels also changes in a periodic manner, leading the electrical signal from the photoelectric sensor to change periodically as the pulse rate.

The main variables in the heart rate monitoring program based on photoelectric volumetric pulse wave tracing method are explained in the Table 1.

The general idea of the program is that the pulse renderer records the voltage waveform signal of the pulse based on the returned light. The program uses QS to determine if a heartbeat is detected. The voltage signal is sampled and converted to a digital signal by a microcontroller, which then sends the signal to the host computer for recording and displaying. The microcontroller calculates the time difference between two adjacent pulse wave crest points, which corresponds to the time interval between two heartbeats, known as the Inter-Beat Interval (IBI).

The microcontroller core program is divided into four main parts: sampling, filtering, calculation, and output [6].

Sampling: The pulse wave sensor output pulse analog signal, which is sampled by the analog-to-digital converter at a rate of 500 Hz.

Filtering: Because of the reflection of the pulse wave in the artery, a strong beat wave is often present. The program is set to track the increase in the pulse once every 0.6 IBI values.

**Figure 1:**
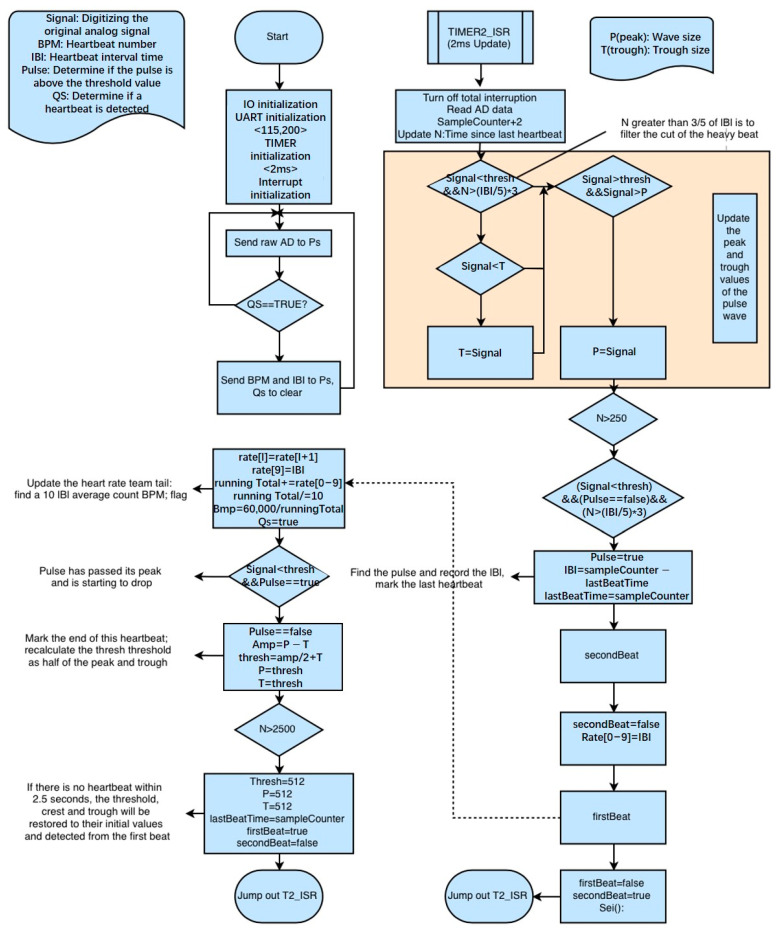
Flow chart of pulse wave sensing signal processing and transmission.

Calculation: Based on the IBI value to calculate the heart rate of meat sheep, resulting in the Beat Per Minute (BPM) value, as shown in Figure 1.

Output: The microcontroller transmits the signal data via a serial protocol. The signal comprises three kinds of data: the prefix “S” represents the pulse wave data; the prefix “B” represents the pulse pressure value; the prefix “Q” represents the IBI value.

(2) Far-infrared thermal perception model

Infrared thermometry [10] is a nondestructive testing technique that measures the temperature of an object by detecting its emitted infrared radiation. The method is characterized by its small size, wide measurement range, high sensitivity, and inherent safety. The principle behind infrared temperature measurement is when the temperature of the object to be measured is higher than absolute zero, charged particles within the object move and produce invisible infrared energy waves [11]. According to Stefan Boltzmann’s law, the absolute temperature can be deduced from the radiation power, radiation coefficient, and proportionality coefficient. The output signal comprises both the temperature of the object being measured and the temperature of the sensor itself.

#### 2.1.2. Data Preprocessing

The extraction of feature information from biosensing signals to calculate heart rate and body temperature relies on a high signal-to-noise ratio. To improve the accuracy of these calculations, it is essential to suppress noise and interference. This section focuses on processing methods designed to effectively eliminate noise interference signals from pulse wave sensing signals. These methods include QRS waveform extraction method [12,13], wavelet threshold noise cancellation method [14], artifact elimination [15,16], and modern spectral estimation to extract feature. 

The transfer function of the low-pass filter is defined as follows:

(1) $$H\left(z\right)=\frac{1}{32}\frac{{\left(1-z^{-6}\right)}^{2}}{{\left(1-z^{-1}\right)}^{2}}$$

Deriving and differentiating this transfer function of the above transfer function leads to the corresponding differential equation:

(2) $$y\left(n\right)=2y\left(n-1\right)-y\left(n-2\right)+\frac{1}{32}\left[x\left(n\right)-2x\left(n-6\right)+x\left(n-12\right)\right]$$

The high-pass filter is constructed from an all-pass filter and a low-pass filter, with its transfer function expressed accordingly.

(3) $$H_{hp}=z^{-16}-\frac{1}{32}\left(\frac{\left(1-z^{-32}\right)}{\left(1-z^{-1}\right)}\right)$$

Similarly, the difference equation obtained through differentiation is presented here.

(4) $$y\left(n\right)=x\left(n-16\right)-\frac{1}{32}\left[y\left(n-1\right)-x\left(n\right)-x\left(n-32\right)\right]$$

The cutoff frequency of the high-pass filter is set at 5 Hz, with a delay of 80 ms. Consequently, the cutoff frequency of the band-pass filter designed to suppress power line interference is 5 to 11 Hz. By performing differential operation on the filtered signal derived from the previous equations, we obtained Equation (5):

(5) $$y\left(n\right)=\frac{1}{8}\left[2x\left(n\right)+x\left(n\right)-x\left(n-3\right)-2x\left(x-4\right)\right]$$

However, the peaks obtained by the operation of the above equation may still exhibit double peaks, necessitating the use of an active window integral of a certain width *N*. For a sampling rate of 200 Hz, *N* = 30 is an appropriate choice. This active window integral is defined as follows:

(6) $$y\left(n\right)=\frac{1}{N}\left\{x\left[n-\left(N-1\right)\right]+x\left[n-\left(N-2\right)\right]+\ldots +x\left(n\right)\right\}$$

After completing the waveform extraction process, a series of wave peaks can be detected.

Since this waveform extraction method is not sufficient to eliminate noisy signals, a detailed classification is required, which generally follows the following principles and procedures:

(1) Filter out all waveforms within 200 ms before and after the larger waveform;
(2) If the detected waveform has no more double peaks than the original signal, this suggests that the original signal may have baseline drift, necessitating artifact denoising;
(3) If the peak of the extracted waveform occurs within 360 ms of the former QRS wave and the peak value is less than half of the amplitude of this QRS waveform, then this extracted waveform is identified as a T-wave, not a QRS wave;
(4) If the peak value of the extracted waveform exceeds the threshold $I_{1}$, it is classified as a QRS waveform; otherwise, it is considered interference noise;
(5) If no QRS waveform greater than 1.5 times the previous right posterior interval of $I_{1}$, is detected, the peak of the previous QRS waveform, occurring 360 ms after the detection threshold $I_{2}$, is considered as a QRS waveform.

(1) Wavelet threshold noise elimination method

The wavelet threshold denoising method [16] is based on the principle of setting an appropriate threshold for the sub-band coefficients derived from the noise distribution. When the wavelet coefficients are below this given threshold, they are deemed to be noise-generated and can either be set to zero or reduced, while coefficients above the threshold should remain unchanged. Wavelet threshold noise elimination employs window functions of varying durations for frequency component differentiation analysis, allowing for the targeted removal of both high- and low-frequency noises. The biosensing device in this experiment contains circuit components such as resistors and diodes, which generate thermal noise and scattered particle noise. Additionally, the third category of excess noise generated in the circuit is particularly at low frequencies. Therefore, filtering out these noises and interferences is an essential step prior to signal analysis and calculation. The method mainly consists of the following steps:

(1) Decomposition: Choose a suitable wavelet basis function and decomposition scale to decompose the noisy signal into target scale layers;
(2) Threshold denoising process: Select a threshold for the sub-band coefficients at each scale obtained from the decomposition, apply a threshold operation to eliminate the target noise, and derive new wavelet coefficients;
(3) Reconstruction: Sequentially reconstruct the new wavelet coefficients processed at each scale to obtain the denoised signal.

**Figure 2:**
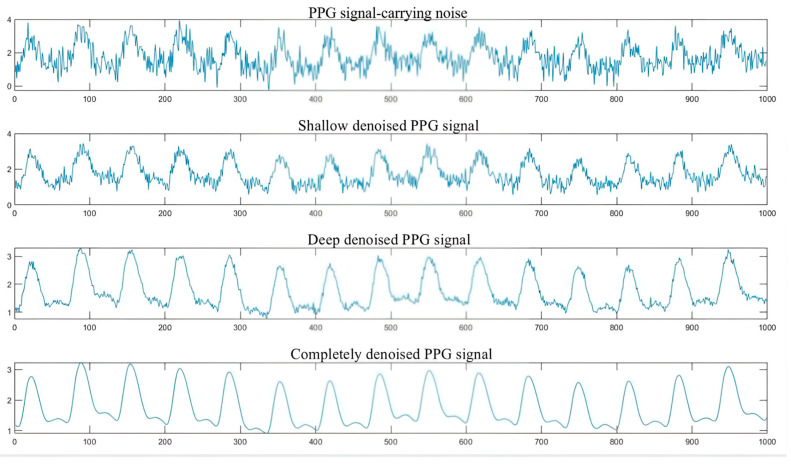
Reconstruct the signal to obtain the PPG signal.

Considering that the wavelet bases should exhibit regularity and symmetry, double orthogonal wavelets are employed to eliminate baseline drift and industrial frequency interference. The pulse wave signal is decomposed into 9 scale wavelets using the Bior6.8 wavelet. The soft threshold function is utilized with the threshold calculated based on the maximum–minimum criterion. The wavelet coefficients for scales 1, 2, and 3 are set to zero, while the low-frequency approximation signal of scale 9 is filtered out. The reconstructed signal obtained from the pulse wave signal is illustrated in Figure 2. As shown in the figure, the denoised pulse wave signal effectively reduces the baseline drift, motion artifacts, and industrial frequency interference.

(2) Modern spectral estimation methods for characteristic parameters.

Parameter estimation is a method for identifying the parameters of characteristic variables from signals affected by disturbances and noise, utilizing signal estimation and statistical machine learning theory [17]. Basically, it is a method of extracting waveform samples from the total signal to estimate unknown parameters contained in the signal distribution. The primary objective of parameter estimation is to extract the characteristic parameters from the noisy signal. In this paper, we estimated and computed the main statistics of the signal based on a set of non-stationary random signal samples obtained through detection [18,19]. The mean and variance of the random variables are estimated as follows:

(7) $${\hat{m}}_{x}=\frac{1}{N}\sum_{i=1}^{N}x_{i}$$

(8) $$ar\left(x\right)=\frac{1}{N}\sum_{i=1}^{N}{\left[x_{i}-E\left(x\right)\right]}^{2}$$

The process of determining the characteristic signal parameters from the signal with noise is shown in Figure 2. The biological observation signal data item *x* is a combination of co-biological signal s and noise interference signal n. This relationship can be expressed as x=s(θ)+n*,* where θ denotes characteristic parameters of the signal. The feature parameter estimation estimates θ based on statistical machine learning algorithm, while optimizing the relationship between θ and θ according to an appropriate criterion. Common optimization methods employed in this context include the minimum mean square error criterion, which seeks to minimize $E\left[{\left(\theta -\hat{\theta }\right)}^{2}\right]$.

#### 2.1.3. Construction of Evaluation Indicators

We established the inference rules for diagnosing abnormal logistics and transportation, as well as the diagnostic discriminative methods by summarizing and generalizing the assessment experience. Knowledge rules were developed from three sources: biologically apparent information, transportation environment information, and biological perception signals This process includes discussions and consultations with domain experts, field investigation, and observation.

Several field studies in Inner Mongolia, Qinghai, Liaoning, and Henan were conducted to gather information on the external apparent stress state of meat sheep during transportation. According to the scoring method of Karolinska sleepiness scale [20], the stress states of meat sheep were quantified into three levels, i.e., comfort level 1, stress level 2, and intermediate state level 3, corresponding to category labels 1, 2, and 3, respectively [9].

### 2.2. Methods

The graded prediction model of transport stress in meat sheep constructed in this paper is a method to reflect the stress level of meat sheep in a certain transport environment by measuring the biosignals of the organism. In this model, the primary focus of the study is the degree of stress response in meat sheep and the establishment of the graded relationship model between biosignals and stress level serves as the means of investigating this stress response. In the model construction, pulse wave signals, heart rate variability parameters, and body temperature characteristics are the input variables and the stress level is the output variable. The constructed stress grading model can indirectly indicate the stress levels of meat sheep through biological and environmental signals, providing a foundational method for assessing their health status, mortality, or readiness for slaughter.

#### 2.2.1. Construction of SVM Model

Support vector machines (SVMs) are a pattern recognition method based on statistical learning theory. The purpose of SVM [21] is to find a hyperplane that maximizes the separation of data points from two classes, while keeping them as far apart as possible from the classification plane. To achieve this, an optimization problem under constraints is usually constructed and solved to obtain a classifier.

Additionally, the support vector machine algorithm [22] has the property of being linearly separable in high-dimensional space, which enables it to address nonlinear problems. Specifically, the kernel function of the algorithm is utilized to map the original spaces to higher dimensional feature spaces, making the problem linearly divisible. Therefore, the radial basis function kernel is chosen as the kernel function for grade classification to solve the nonlinear transportation stress grading prediction problem of sheep. In order to achieve a higher recognition rate and speed, we adopted a one-vs.-all strategy to split the multiclassification problem into multiple binary classification problems. The kernel function used in this study is a linear kernel function.

The linear kernel function is as follows:

(9) $$k\left(x_{i},x_{j}\right)={x_{i}}^{T}x_{j}$$

#### 2.2.2. Construction of GBDT Model

GBDT (Gradient Boosting Decision Tree) [23,24] is one of the most effective algorithms for fitting true distribution and can be employed for classification, regression, and features filtering. It performs multiple iterations, with each iteration producing a weak classifier, typically chosen as a Classification and Regression Tree (CART). The regression tree is trained on the residuals of the previous classifiers. Weak classifiers are generally simple, exhibiting low variance and high bias. This is because the training process aims to improve the accuracy of the final classifier by reducing the bias.

(1) Initialization into the weak learner
  (10) $$f_{0}\left(x\right)=arg{min}_{c}\sum_{i=1}^{N}L\left(y_{i},c\right)$$
(2) Form = 1,2,…,M, there are:
  (1) Calculate the residuals for each sample i=1,2,…,N
    (11) $$r_{im}=-{\left[\begin{array}{c} \partial L\left(y_{i},f\left(x_{i}\right)\right)\\ \partial f\left(x_{i}\right) \end{array}\right]}_{f\left(x\right)=f_{m-1}\left(x\right)}$$
    The residual obtained in the previous step is used as the new true value of the sample, and the data ($x_{i},r_{im}$), i=1,2,…,N, serve as the training data for the next tree, resulting in a new regression tree $f_{m}(x)$, whose corresponding leaf node region is $R_{im}$. Here, j=1,2,…,J and *J* represents the number of leaf nodes in the regression tree.
  (2) For the leaf region j=1,2,…,J calculate the best-fit value
    (12) $$r_{jm}=argmin\sum_{x_{i}\in R_{jm}}L\left(y_{i},f_{m-1}\left(x\right)+r\right)$$
  (3) Update the learner
    (13) $$f_{m}\left(x\right)=f_{m-1}\left(x\right)+\sum_{j=1}^{J}r_{jm}I\left(x\in R_{jm}\right)$$
    (14) $$f\left(x\right)=f_{M}\left(x\right)=f_{0}\left(x\right)+\sum_{m=1}^{M}\sum_{j=1}^{J}r_{jm}I\left(x\in R_{jm}\right)$$
  (4) Obtain the final learner
    The primary advantage of GBDT is the flexibility to handle various types of data, including continuous and discrete values. Additionally, GBDT often achieves a higher level of predictive accuracy with comparatively less tuning time than Support Vector Machines (SVMs). Furthermore, by employing robust loss functions, GBDT demonstrates strong resilience to outliers.

#### 2.2.3. Construction of XGB Model

XGB (Extreme Gradient Boosting) is an implementation of the boosting algorithm [25]. XGB focuses on reducing the bias and improving model accuracy by utilizing multiple weak learners, each of which is relatively simple. To avoid overfitting, the next learner learns the difference between the result of the previous base learner and the actual value. By learning multiple learners, the overall discrepancy between the predictions and the actual values is progressively minimized.

(15) $${y_{i}}^{0}=0$$

(16) $${y_{i}}^{1}=f_{1}\left(x_{i}\right)={y_{i}}^{0}+f_{1}\left(x_{i}\right)$$

(17) $${y_{i}}^{2}=f_{1}\left(x_{i}\right)+f_{2}\left(x_{i}\right)={y_{i}}^{1}+f_{2}\left(x_{i}\right)$$

(18) $${y_{i}}^{t}=\sum_{k=1}^{t}f_{k}\left(x_{i}\right)={y_{i}}^{t-1}+f_{t}\left(x_{i}\right)$$

The fundamental concept is to generate new trees, with each tree learned based on the difference between the previous tree and the target value, so that the bias of the model can be reduced. The results of all trees are accumulated to be the predicted value of the model. It is up to the objective function to decide how to generate a better tree at each step. In XGB, the objective function takes the following form:

(19) $$Obj\left(\theta \right)=L\left(\theta \right)+\Omega \left(\theta \right)$$

The objective function consists of two parts: L(θ) represents the loss function, i.e., the model error, which is the difference between the true value of the sample and the predicted value. The commonly used loss functions are squared loss and logistic loss. Ω(θ) represents the regularization term, the model’s structural error, which is used to constrain the complexity of the model.

## 3. Results

The input parameters of the model include PPG signal characteristics related to transport stress and body temperature signal characteristics. PPG signal characteristics consist of the heart rate variability parameters HR, MR, SDNN, RMSSD, pNN50, and CV. Body temperature signal characteristics are the TO. The output of the model is the predicted levels of stress assessment in meat sheep. Regarding the classification of transport stress states in the knowledge rule, the stress states of meat sheep were categorized into three levels, comfort level 1, stress level 2, and intermediate state level 3, corresponding to category labels 1, 2, and 3, respectively.

### 3.1. Model Parameter Input

After QRS waveform extraction, wavelet threshold noise elimination, and modern spectral estimation, the PPG signal and body temperature signal were combined with expert qualitative evaluation of transport stress. The aim of normalizing the bio-perceptual feature, parameters of transport stress of meat sheep, was to scale them to the range of [0, 1]. We obtained 195 sets of valid data samples of PPG and body temperature signals during seven hours, and each set of samples contained seven-dimensional feature parameters. Among them, 136 sets are training set and 59 sets are test sets.

### 3.2. Model Training

(1) SVM model implementation

Basing on the SVM principle, the construction of a transport stress hierarchical prediction model consists of three typical steps. During the algorithmic model input variable selection, input sets consist of pulse wave signals, heart rate variability parameters, and body temperature characteristic parameters. In the model training phase, the kernel functions are adjusted according to the requirements. Different algorithms are employed to optimize the model tuning parameters required for model operation, and the corresponding transport stress hierarchical prediction model is obtained after algorithmic training. In the testing phase, the model utilized the test set to evaluate the accuracy of the established transport stress classification prediction.

Two key parameters can be set in the SVM algorithm model: the loss factor c and the kernel function selection factor g. The loss factor c is the inverse of the weight of the penalty term. A smaller value indicates a stronger penalty term, causing the model to focus more on the data points near the center of the category, which is called soft margin. Conversely, when c is too large, the model is more sensitive to the anomalies near the separation hyperplane, which is known as the hard margin. In this section, we utilized a third-party library scikit-learn to build the model and employed the automatic parametric method Grid Search to find the most appropriate model. Grid Search involves multiple kernel function labels for linear kernel functions, Gaussian kernel functions, polynomial kernel functions, and sigmoid kernel functions. They are called consecutively to forecast the transport stress classification of meat sheep.

(2) GBDT model implementation

The key parameter in the GBDT algorithm model is n_estimators. N_estimators is the maximum number of the weak learner iterations. When n_estimators is too small, there is a risk of underfitting, while when n_estimators is too large, there is a risk of overfitting. Generally, a moderate value should be chosen. The default value is 100. N_estimators is considered along with the parameter learning rate, which is set to 300. Learning_rate, the weight reduction factor ν for each weak learner, also known as step size, is set to 0.1. Since it is used as the classification model, the default “deviance” is used. No weight is considered, so min_weight_fraction_leaf is 0. The mode is better optimized for binary separation and multivariate classification.

(3) XGB Model Implementation

The XBG algorithm model has three key parameters that can be set: the generic parameter, the Booster parameter, and the learning target parameter. The Booster parameter controls the booster at each step. The Booster parameter generally regulates the effect of the model and the computational cost. The two main models used are gbtree and gblinear. Gbtree utilizes a tree-based model for boosting computation, while gblinear employs a linear model for boosting computation. The learning target parameters govern the performance of the training objectives. Our classification of the problem is primarily based on these learning target parameters.

The transport stress classification prediction is a multi-classification problem. In order to obtain a higher recognition rate and speed, the gbtree model is used. XGBoost is set to perform multi-classification using the softmax objective function. Given the three levels of the stress status of meat sheep, the parameter num_class is set to 3. Max_depth is the depth of the tree, and the larger its value, the easier it is to overfit; the smaller its value, the easier it is to underfit. Max_depth 12 is set in this study.

Min_child_weigh specifies the minimum sample weight sum of the child nodes. If the sample weight sum of a leaf node is smaller than min_child_weight, the splitting process is finished and min_child_weigh is set to 3. Gamma penalty term coefficient determines the minimum loss function drop required for node splitting. The larger the value of this parameter, the more conservative the algorithm is. In this research, gamma is set to 0.1.

### 3.3. Model Parameter Design and Training

**Figure 3:**
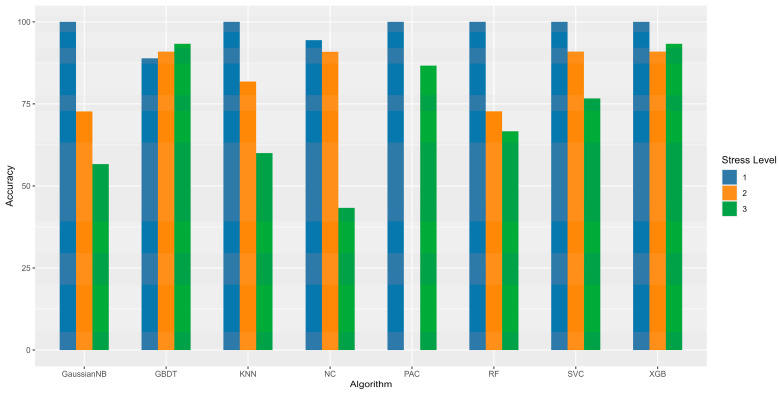
Comparison figure of classification accuracy of different machine learning algorithms for optimization level.

To assess the precision of the trained model, a test set of 59 sets of data was used with optimal parameters to evaluate the overall performance of the graded prediction model for the transport stress assessment of meat sheep. The classification results of the test set are displayed in Table 2 [26,27] and Figure 3.

### 3.4. Evaluation of Classification Results

The eight meat sheep transportation stress classification prediction models trained in the previous step were evaluated on the test set data samples. According to the model test results, the accuracy of different sets is shown. The models trained by the three algorithms SVC, GBDT, and XGB demonstrate average prediction accuracy exceeding 80%. SVC, XGB achieved the highest prediction accuracy of 100% for stress level 1, and GBDT, XGB reached the highest prediction accuracy of 93.33% for stress level 3. The XGB algorithm with the best results was selected and predicted accuracies of 100% for level 1, 90.91% for level 2, and 93.33% for level 3, resulting in an overall accuracy of 94.92%.

The data in the table indicate that most models have a higher classification accuracy in level 1 and level 2 compared to level 3, which has a certain chance of misclassification results. Combined with the actual situation of the meat sheep transportation process, it can be concluded that level 1 and level 2 represent two more extreme states for meat sheep. That is, comfortable non-stress and definite stress. Since the pulse wave and body temperature signal characteristics of the meat sheep dealing with these two states are easily recognizable, the prediction accuracy of the stress model is high. However, if the meat sheep are in a state of mild stress, the pulse wave and body temperature signal characteristics may not change significantly or may carry less weight at this stage, resulting in a slightly lower prediction rate for the overall model. Moreover, the experimental design could not exclude model misclassification due to physical or neurological differences among test sheep.

## 4. Conclusions

In this paper, we designed experiments to obtain biological sensing signals and environmental monitoring parameters of meat sheep transportation to measure the stress changes of meat sheep during field transportation. We took heart rate, heart rate variability, and body surface temperature as the predictors of transportation stress in meat sheep. Then, we used the QRS waveform extraction method, wavelet threshold noise elimination method, and modern spectral estimation to extract feature information method to effectively eliminate noise interference in pulse wave sensing signals.

Based on the machine learning theory, a transport stress level evaluation model was established based on the extraction of PPG and body temperature signal feature parameters. HRV key indicators HR, MR, SDNN, RMSSD, pNN50, and CV were used as PPG signal feature parameters. The body temperature characteristic signal ST was utilized as the characteristic parameter of body temperature signal. The PPG signal feature parameters and body temperature signal feature parameters were used as input parameters for each machine learning algorithm. The transport stress levels included comfort level 1, stress level 2, and intermediate state level 3 as the output parameters. The accuracies of three optimized parameters were 100% for level 1, 90.91% for level 2, and 93.33% for level 3, resulting in an overall accuracy of 94.92%.

In conclusion, the model to evaluate meat sheep transport stress status was constructed by the training the polar gradient boosting tree model. Using the characteristic parameters of biosensing signals PPG and body temperature, the model demonstrates strong performance and high accuracy in grade recognition [28]. It confirmed that the wearable biosensing dynamic detection system created in this research can accurately monitor and quantitatively evaluate biological signals and environmental data while transporting meat sheep. The system enhances the efficiency of managing sheep transportation, decreases loss risks, cuts transportation expenses, improves animal health during transportation, and boosts the economic gains of meat sheep transportation [29]. For further application, the wearable biosensing detection device created in this research can be tested for compatibility with different species to explore its detection effectiveness when used with other livestock like pigs and cattle [30], broadening its potential applications.

## Figures and Tables

**Table 1:** Description of the main variables of the pulse wave monitoring program.

	Refresh Time	Variable Description
Signal	2 ms	Unprocessed pulse wave sensor signal
IBI	per beat	Beat interval time (milliseconds)
BPM	per beat	Number of beats per minute
QS	Initial value of each heartbeat	User to clear
Pulse	Initial value of each heartbeat	Cleared by ISR function

**Table 2:** Comparison table of classification accuracy of different machine learning algorithms for optimization level.

Algorithm	Stress Level	Prediction Accuracy/Test Set	Rank Accuracy (%)	Total Accuracy (%)
Gaussian Naive Bayes (Gaussian NB)	1	18/18	100.00	72.88
	2	8/11	72.72	
	3	17/30	56.67	
Passive-Aggressive Classifier (PAC)	1	18/18	100.00	74.58
	2	0/11	0.00	
	3	26/30	86.67	
Nearest Centroid (NC)	1	17/18	94.44	67.80
	2	10/11	90.90	
	3	13/30	43.33	
KNN	1	18/18	100.00	76.27
	2	9/11	81.82	
	3	18/30	60.00	
RF	1	18/18	100.00	77.97
	2	8/11	72.73	
	3	20/30	66.67	
SVC	1	18/18	100.00	86.44
	2	10/11	90.91	
	3	23/30	76.67	
GBDT	1	16/18	88.89	91.53
	2	10/11	90.91	
	3	28/30	93.33	
XGB	1	18/18	100.00	94.92
	2	10/11	90.91	
	3	28/30	93.33	

## Data Availability

Data are contained within the article.
